# Parkin-mediated ubiquitination of mutant glucocerebrosidase leads to competition with its substrates PARIS and ARTS

**DOI:** 10.1186/1750-1172-9-86

**Published:** 2014-06-16

**Authors:** Inna Bendikov-Bar, Debora Rapaport, Sarit Larisch, Mia Horowitz

**Affiliations:** 1 Department of Cell Research and Immunology, Life Sciences, Tel Aviv University, Levanon St, Ramat Aviv 69978, Israel; 2 Department of Human Biology, Natural Sciences, Haifa University, Haifa, Israel

**Keywords:** Gaucher disease, Parkinson disease, Glucocerebrosidase, Paris, Arts

## Abstract

**Background:**

Parkinson’s disease (PD) is a movement neurodegenerative disorder characterized by death of dopaminergic neurons in the substantia nigra pars compacta of the brain that leads to movement impairments including bradykinesia, resting tremor, postural instability and rigidity. Mutations in several genes have been associated with familial PD, such as parkin, pink, DJ-1, LRKK2 and α-synuclein. Lately, mutations in the GBA gene were recognized as a major cause for the development of PD.

Mutations in the GBA gene, which encodes for lysosomal β-glucocerebrosidase (GCase), lead to Gaucher disease (GD), an autosomal recessive sphingolipidosis characterized by accumulation of glucosylceramide, mainly in monocyte-derived cells. It is a heterogeneous disease, with Type 1 patients that do not present any primary neurological signs, and Type 2 or Type 3 patients who suffer from a neurological disease. The propensity of type 1 GD patients and carriers of GD mutations to develop PD is significantly higher than that of the non-GD population.

We have shown in the past that parkin and mutant GCase, expressed in heterologous systems, interact with each other, and that normal but not mutant parkin mediates K48-dependent proteasomal degradation of mutant GCase variants.

**Methods:**

We tested possible competition between mutant GCase and PARIS or ARTS on the E3 ubiquitin ligase parkin, using coimmunoprecipitation assays and quantitative real-time PCR.

**Results:**

We show that endogenous mutant GCase variants associate with parkin and undergo parkin-dependent degradation. Mutant GCase competes with the known parkin substrates PARIS and ARTS, whose accumulation leads to apoptosis. Dopaminergic cells expressing mutant GCase are more susceptible to apoptotic stimuli than dopaminergic cells expressing normal GCase, present increased cleavage of caspase 3 and caspase 9 levels and undergo cell death.

**Conclusions:**

Our results imply that presence of mutant GCase leads to accumulation of parkin substrates like PARIS and ARTS, which may cause apoptotic death of cells.

## Background

Gaucher disease (GD) is an autosomal recessive disease characterized by accumulation of glucosylceramides, mainly in monocyte derived cells. Due to its heterogeneity, it has been divided to Type 1 GD, primarily a non-neurological disease, and Type 2 and 3, two forms associated with a neuronopathic disease [1,2]. Mutant glucocerebrosidase (GCase) variants undergo ER-associated degradation (ERAD), the degree of which correlates with disease severity [3]. ER-retained mutant GCase leads to ER stress and to unfolded protein response [4].

There is a significantly higher propensity of GD patients or carriers of GD mutations to develop Parkinson’s disease (PD) compared to the general population [5-19], indicating that not only GD pathology but even the presence of one mutant GBA allele increases the risk for the development of PD.

PD, the second most common neurodegenerative disorder, is characterized by a progressive loss of dopaminergic neurons in the substantia nigra pars compacta of the midbrain [20,21]. It is also associated with the appearance of Lewy bodies (composed of α-synuclein and ubiquitin), depletion of striatal dopamine and gliosis. PD pathology also manifests in non-dopaminergic nerve cells such as olfactory and brain stem neurons, and actually precedes the pathological changes seen in dopaminergic neurons [22]. Motor symptoms in patients, including slowness of movement, resting tremor, rigidity, postural instability and gait difficulty, usually appear after a massive loss of dopamine from the striatum [23]. PD patients may also present non-motor manifestations such as dementia and psychiatric symptoms [24].

GD is associated with glucosylceramide accumulation and pathogenic presence of mutant GCase, yet it is still unclear whether both contribute to development of PD in GD patients and carriers of GD mutations. One theory suggests a role for glucosylceramide accumulation in α-synuclein aggregation [25]. However, there are no documented reports on accumulation of glucosylceramide in brains of Type 1 GD patients, nor in brains of carriers of GD mutations, raising the possibility that the development of PD in GD patients or in carriers of GD mutations is due to the presence of mutant GCase.

We have shown in the past that mutant GCase undergoes parkin-mediated K48 polyubiquitination and proteasomal degradation. Parkin, a multifunctional RING (Really InterestiNG) domain-containing E3 ubiquitin ligase encoded by the PARK2 gene is a cytoplasmic protein, mutations in which are the most common cause (~50%) of autosomal recessive PD [26,27]. Parkin regulates diverse functions in the cell, including receptor-mediated trafficking, cell signaling, autophagy and mitochondrial quality control [28]. Parkin regulates receptor-mediated trafficking and signaling through monoubiquitination of substrates [29]. It regulates inclusion body formation and autophagy through lysine 29 and 63 polyubiquitination [30,31]. Parkin also mediates lysine 48 polyubiquitination of substrates, which are destined to proteasomal degradation [28,32]. Loss-of-function mutations in the PARK2 gene lead to accumulation of non-cleared lysine-48 substrates. Accumulation of the known parkin substrates PARIS [33] and ARTS [34] is pathogenic to cells. Such accumulation was found in post mortem brains of PD patients and in a mouse model of PD mutated in PARK2 [35], indicating that the loss of parkin function contributes to PD pathogenesis.

In this study we used GD-derived skin fibroblasts to show that endogenous mutant GCase associates with parkin and undergoes parkin-dependent degradation. We utilized human dopaminergic cells in tissue culture to demonstrate competition between mutant GCase and the known parkin substrates PARIS and ARTS, whose accumulation leads to apoptosis of cells.

## Material and methods

### Materials

The following antibodies were used in this study: monoclonal anti-GCase 2E2 (Abnova), generated against a peptide corresponding to amino acids 146–236 of human GCase, rabbit polyclonal anti-GCase, generated against a peptide corresponding to amino acids 517–536 of human GCase (G4171, Sigma), rabbit anti-ERK (C16 Santa Cruz Biotechnology, Santa Cruz, CA, USA), mouse monoclonal anti-actin (MP Biomedicals, OH, USA), mouse monoclonal anti-myc and anti-GFP (Cell Signaling Technology, Beverly, MA, USA); Secondary antibodies: horseradish peroxidase-conjugated goat anti-mouse or goat anti-rabbit (Jackson Immuno Research Laboratories, West Grove, PA, USA), rabbit anti-caspase 3 and rabbit anti-cleaved caspase 9 (Cell Signaling Technology, Beverly, MA, USA).

Carbobenzoxy-L-leucyl-L-leucyl-L-leucinal (MG132), cyclohexamide (CHX), Leupeptin and phenylmethylsulfonyl fluoride (PMSF) were purchased from Sigma-Aldrich (Rehovot, Israel). Four-methyl-umbelliferyl-glucopyranoside (4-MUG) was purchased from Genzyme Corp (Cambridge, MA, USA). Nonidet P-40 (NP-40) was purchased from Roche Diagnostics (Mannheim, Germany). Absolute Blue qPCR SYBR Green ROX Mix was from TAMAR Laboratory Supplies (Mevaseret Zion, Israel).

### Cell lines

Human primary skin fibroblasts cell lines are described in Table 1. Fibroblasts and SHSY5Y human dopaminergic cells were grown in Dulbecco’s modified Eagle’s medium (DMEM) supplemented with 20% fetal calf serum (FCS), 100 U/ml penicillin-streptomycin, 1 mM sodium pyruvate and 2 mM L-glutamine (Biological Industries, Beit-Haemek, Israel), at 37°C in the presence of 5% CO_2_. SHSY5Y stably expressing different human GCase variants were described elsewhere [36].

**Table 1:** Description of cell lines used in the study

**Patient #**	**Genotype**	**Disease type**	**Age (years)**	**Sex**
1	Normal	Normal	57	F
2	Normal	Normal	UN*	UN*
3	L444P/L444P	3	1	F
4	L444P/L444P	3	1	F
5	L444P/L444P	3	2	F
6	L444P/L444P	3	3	F
7	L444P/RecNci	2	1	M
8	N370S/N370S	1	43	F
9	R131C/R131C	2	1 month	F
10	L444P/R120W	2	1	F
11	L444P/P415R	2	11 months	F
12	N370S/N370S	1(severe)	11	M
13	N370S/V394L	1	30	M
14	K157Q/D140H_E326K	1	26	M
15	K157Q/D140H_E326K	1(severe)	13	M

The table contains information on genotype, GD type, sex and age at diagnosis of the GD-derived fibroblast lines and normal fibroblasts. *UN-Unknown (biopsy was taken anonymously due to Israeli Helsinki requirements, with no tracking back information).

### Construction of plasmids

Construction of myc-His-GCase plasmids was described elsewhere [36]. pSC2-6myc ARTS was described in [34]. MISSION shRNA plasmid (TRCN0000000285) encoding parkin-targeted shRNA was purchased from Sigma-Aldrich (St Louis, Mo, USA).

To construct the PARIS-expressing vector, a 1.9 kb PARIS cDNA fragment was amplified using Phusion high-fidelity DNA polymerase (New England Biolabs, Ipswich, USA) from the plasmid cFUGW-lenti-PARIS, a kind gift from Dr. Ted M. Dawson (Solomon H. Snyder Department of Neuroscience, Johns Hopkins University School of Medicine, Baltimore, Maryland, USA). The PARIS cDNA fragment was cloned into the *Ecl136II* site of pEGFPC3 vector plasmid (Clontech Laboratories Inc. CA, USA). Gibson assembly technology (New England Biolabs, Ipswich, USA) was used for the cloning. For knockdown of parkin, MISSION short hairpin RNA (shRNA) plasmids, encoding small interfering RNAs (siRNAs) targeting parkin, were purchased from Sigma Aldrich (St Louis, Mo, USA). Of all the existing vectors, TRCN0000000285 successfully knocked down human parkin. As a control, a pLKO.1 plasmid (Sigma Aldrich, St Louis, Mo, USA) harboring shRNA against GFP was used.

### RNA preparation

Total RNA was isolated using the EZ-RNA kit (Biological Industries, Beit Haemek, Israel), according to the manufacturer’s instructions.

### RT PCR

Two micrograms of RNA were reverse transcribed with M-MLV reverse transcriptase (Promega corporation, CA, USA), in the presence of 1 μg oligo-dT primer in a total volume of 20 μl, at 42°C for 60 minutes. Reactions were stopped by incubation at 70°C for 15 minutes. One-two microliters of the resulting cDNA were amplified by quantitative real-time PCR.

### Quantitative real-time PCR

One microliter of cDNA was used for real-time PCR. PCR was performed using the KAPA SYBR Fast Universal qPCR kit (Kapa Biosystems, Wilmington, MA, USA) in a Rotor-Gene 6000 (Corbett life sciences, Valencia, CA, USA). The reaction mixture contained 50% qPCR mix, 300 nM of forward primer (5′-ATCTGAAGGAGCAACATCTGG-3′) and 300 nM of reverse primer (5′-CACGGGCGAGTTTACTATGTAG-3′), in a final volume of 10 μl. Thermal cycling conditions were: 95°C (10 minutes), 40 cycles of 95°C (10 seconds), 60°C (20 seconds) and 72°C (20 seconds). Relative gene expression was determined by Ct value.

### SDS-PAGE and western blotting

Cell monolayers were washed three times with ice-cold phosphate-buffered saline (PBS) and lysed at 4°C in 500 μl of lysis buffer (10 mM HEPES pH 8.0, 100 mM NaCl, 1 mM MgCl_2_ and 1% Triton X-100) containing 10 μg/ml aprotinin, 0.1 mM PMSF and 10 μg/ml leupeptin. Lysates were incubated on ice for 30 minutes and centrifuged at 10,000 *g* for 15 minutes at 4°C. Samples, containing the same amount of protein, were electrophoresed through 10% SDS-PAGE and electroblotted onto a nitrocellulose membrane (Schleicher and Schuell BioScience, Keene, NH, USA). Membranes were blocked with 5% skim milk and 0.1% Tween-20 in Tris-buffered saline (TBS) for 1 hour at room temperature (RT) and incubated overnight with the primary antibody. The membranes were then washed three times in 0.1% Tween-20 in TBS and incubated with the appropriate secondary antibody for 1 hour at RT. After washing, membranes were reacted with ECL detection reagents (Santa Cruz Biotechnology Inc., CA, USA) and analyzed by luminescent image analyzer (X-OMAT 2000 Processor, Kodak, Rochester, NY, USA).

### Transfections

SHSY5Y cells were transfected using either a MP-100 Microporator (Digital Bio Tech, Seoul, South Korea) according to the manufacturer’s instructions, or Lipofectamine 2000™ (Invitrogen, CA, USA).

### Immunoprecipitation

Subconfluent skin fibroblasts were treated overnight with 25 μM MG-132, after which cells were washed 3 times with ice-cold PBS and lysed at 4°C in 1 ml of lysis buffer (10 mM Hepes pH = 8, 100 mM NaCl, 1 mM MgCl_2_, and 0.5% NP-40) containing 10 μg/ml aprotinin, 0.1 mM PMSF and 10 μg/ml leupeptin (Sigma-Aldrich, Rehovot, Israel). Following centrifugation at 10,000 *g* for 15 minutes at 4°C, the supernatants were pre-cleared for 2 hours at 4°C with protein A-agarose (Roche Diagnostic, Mannheim, Germany). Following washes with 1 ml of lysis buffer containing protease inhibitors, proteins were eluted for 10 minutes at 100°C with 5X loading buffer, electrophoresed through 10% SDS-PAGE and blotted. The corresponding blot was interacted with the appropriate antibodies.

### Ubiquitination in tissue culture

Sub-confluent skin fibroblasts were treated overnight with 25 μM MG-132, after which stringent immunoprecipitation conditions were applied as previously described [37], with some modifications. The medium was aspirated and the cells were harvested and lysed in 100 μl of denaturing buffer (1% SDS, 50 mM Tris pH7.4, 140 mM NaCl). Following vigorous vortexing, the lysates were immediately boiled for 10 minutes, cleared by centrifugation at 10,000 *g* for 10 minutes and diluted 10 fold into buffer containing 2% Triton X-100, 50 mM Tris pH7.4, 140 mM NaCl. After additional centrifugation to remove any insoluble material, immunoprecipitation was carried out with monoclonal anti-GCase antibody. Following three washes (5% sucrose, Tris pH7.5, 50 mM NaCl, 0.5% NP40, EDTA 5 mM) the ubiquitin-GCase conjugates were resolved through SDS-PAGE and the corresponding blots were interacted with polyclonal anti-GCase antibodies.

### Proteasome inhibition

Cells were treated for 24 hours with 25 μM MG132, after which they were processed according to the experiment performed (western blotting with or without immunoprecipitation).

### Quantification

The blots were scanned using Image Scan and the intensity of each band was measured by the Image Master 1DPrime densitometer (both from Amersham Pharmacia Biotech, Buckinghamshire, England). To quantify the results, the intensity of the tested protein at each lane was divided by the intensity of the loading control (actin or ERK) in the same lane. The value obtained for non-transfected cells or WT cells was considered as 1.

## Results

### Endogenous mutant GCase undergoes polyubiquitination and interacts with parkin

**Figure 1:**
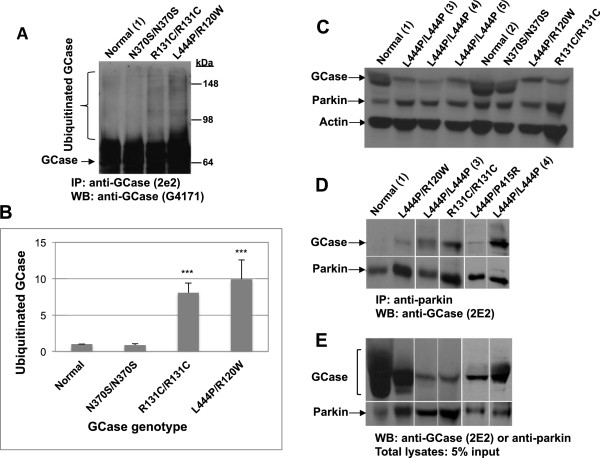
**Mutant GCase undergoes ubiquitination and associates with parkin. (A)** Protein lysates were prepared from MG-132-treated GD-derived skin fibroblasts or normal skin fibroblasts under denaturing conditions. GCase was immunoprecipitated with monoclonal anti-GCase antibody and subjected to western blot analysis and interaction with polyclonal anti-GCase antibodies. **(B)** Densitometry was used to quantify the level of ubiquitination. To normalize the results, intensity of ubiquitinated GCase in each lane was divided by the intensity of total GCase in the same lane. The value obtained for normal cells was considered as 1. The results represent the mean ± SEM of 3–6 independent experiments. ***P < 0.001 in Student *t*-test*.***(C)** Lysates were prepared from MG-132-treated GD-derived skin fibroblasts or from normal skin fibroblasts. They were subjected to western blot analysis and interaction with anti-GCase antibody, anti-parkin antibody, or anti-actin antibody as a loading control. **(D)** Parkin-containing complexes were immunoprecipitated from lysates of MG-132-treated GD-derived skin fibroblasts or normal skin fibroblasts with anti-parkin antibody. They were subjected to western blot analysis and interaction with anti-GCase antibody. **(E)** For loading control, 5% of lysates were subjected to SDS-PAGE and western blot analysis with anti-GCase or anti-parkin antibodies. Patients’ details appear in Table 1. Numbers in parenthesis denote patient-derived cell lines, shown in Table 1.

We have already shown that the L444P mutant GCase variant undergoes ubiquitination [38]. In order to confirm ubiquitination of other mutant variants, GCase-containing complexes were immunoprecipitated from lysates that originated from fibroblasts of GD patients, separated through SDS-PAGE and the corresponding blot was interacted with anti-GCase antibodies. As presented in Figure 1A and B, there was a visible ubiquitination of severe mutant GCase variants, while ubiquitination of the N370S mutation was non visible, as expected for a mild mutation [2,39]. It is worth mentioning that the N370S homozygous cells were derived from a mild patient (patient no. 8, Table 1).

### Parkin interacts with mutant GCase in GD fibroblasts

We have shown in the past that parkin and mutant GCase, expressed in heterologous systems, interact with each other. Moreover, parkin mediates polyubiquitination and proteasomal degradation of mutant, but not normal transfected GCase [36]. In the present study, we aimed at confirming the interaction between endogenous parkin and mutant GCase. In order to do so, we first set out to demonstrate the expression of parkin in skin fibroblasts (Figure 1C). At the next stage, parkin-containing complexes were immunoprecipitated from lysates prepared from GD skin fibroblasts and from normal skin fibroblasts, after which the corresponding blot was interacted with anti-GCase antibody. The results presented in Figure 1D and E show that mutant GCase, but not its normal counterpart, interacted with parkin.

### Parkin mediates degradation of endogenous mutant GCase

**Figure 2:**
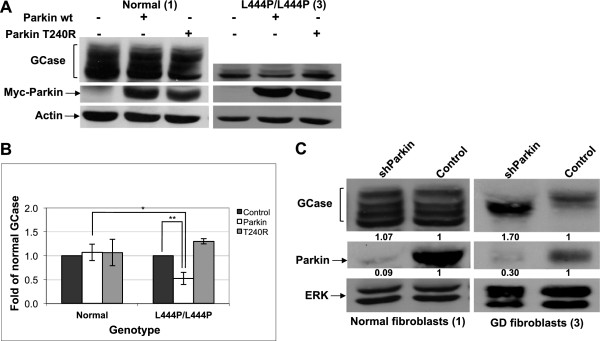
**Parkin mediates degradation of mutant GCase. (A)** Skin fibroblasts from a GD patient or normal skin fibroblasts were transfected with wt or mutant myc-parkin (T240R). Forty-eight hours later cell lysates were prepared and subjected to western blot analysis. The corresponding blot was interacted with anti-GCase, anti-myc and anti-actin antibodies, as a loading control. **(B)** To normalize the results, the blots were scanned and GCase intensity at each lane was divided by the intensity of actin. The value obtained for non-transfected cells was considered as 1. The results represent the mean ± SEM, of three independent experiments. *P < 0.02, **P < 0.01 in Student *t*-test*.***(C)** Skin fibroblasts from a normal individual or from a GD patient were transfected with shRNA containing plasmid designed to down-regulate parkin expression (shParkin) and pLKO.1 plasmid harbouring shRNA against GFP as a control (control). Forty-eight hours later, cell lysates were prepared and subjected to western blot analysis. The corresponding blot was interacted with anti-parkin, anti-GCase, and anti-ERK antibodies as a loading control. To quantify the results, the blots were scanned and GCase or parkin intensity at each lane was divided by the intensity of ERK1/2 in the same lane. The value obtained for control (in normal or GD cells) was considered as 1. The numbers appear below the blots. The cells used in this experiment derived from patient no. 3 and normal individual no. 1 (see Table 1).

To confirm that parkin not only interacts with endogenous GCase but also mediates its degradation, the effect of overexpression of normal or mutant parkin (T240R ligase dead parkin) was tested in skin fibroblasts that originated from GD patient, homozygous for the L444P mutation, or in normal skin fibroblasts. The results, presented in Figure 2A and B, indicated that overexpression of normal, but not mutant, parkin in cells that originated from GD patient led to a significant decrease in GCase level. However, the level of GCase in normal skin fibroblasts was not affected by parkin overexpression. These results strongly suggest that parkin mediates degradation of mutant but not normal GCase.To further confirm that parkin mediates degradation of mutant GCase, normal skin fibroblasts or GD-derived skin fibroblasts were transfected with shRNA directed against endogenous parkin. As shown in Figure 2C, down-regulation of parkin led to stabilization of mutant GCase in GD-derived fibroblasts originated from patient but not in control fibroblasts, indicating that parkin mediates degradation of mutant GCase.

### Competition between PARIS and mutant GCase

As shown thus far, parkin interacts with mutant GCase and mediates its degradation. We showed in a previous study that parkin, expressed in HEK293 cells, mediates K48 polyubiquitination and proteasomal degradation of mutant, but not normal, transfected GCase [36]. Since PD is characterized by death of dopaminergic neurons in the substantia nigra pars compacta region of the brain, and accumulation of pro-apoptotic parkin substrates have been documented, we wondered whether a competition exists between known pathogenic parkin substrates and mutant GCase.

**Figure 3:**
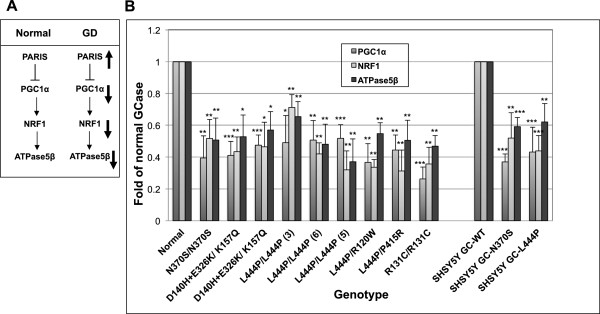
**mRNA level of genes modulated by PARIS is down-regulated in GD fibroblasts or SHSY5Y cells expressing mutant GCase. (A)** Under normal conditions, PARIS represses the expression of PGC1α, which leads to transcriptional inhibition of NRF1 and as a result also of ATPase5β. In GD derived cells, due to competition of PARIS with mutant GCase on parkin availability, a larger fraction of PARIS shuttles to the nucleus. As a result, there is a marked decrease in transcription of PGC1α, NRF1 and ATPase5β. **(B)** mRNA was extracted from normal or GD-derived fibroblasts and from SHSY5Y cells expressing normal or mutant GCase variants. It was subjected to quantitative real-time PCR analysis using primers specific for PGC1α, NRF1 or ATPase5β. The mRNA levels of PGC1α, NRF1 and ATPase5β are presented as a fold of decrease compared to that detected for normal fibroblasts. Values represent the mean ± SEM of 3–7 independent experiments. *P < 0.05, **P < 0.005, ***P < 0.001 in Student *t*-test*.* Numbers in parenthesis denote cell lines detailed in Table 1. Normal represents the average of cell lines 1 and 2 (Table 1).

One of the substrates of parkin is PARIS (PArkin Interactiong Substrate, ZNF746). PARIS undergoes parkin-dependent polyubiquitination and proteasomal degradation. Non-ubiquitinated PARIS shuttles to the nucleus where it serves as a major transcriptional repressor of PGC1α (peroxisome proliferator-activated receptor gamma [PPARγ] coactivator-1α). The latter is a master regulator of mitochondrial biogenesis through induction of NRF1 (nuclear respiratory factor-1) gene expression [33]. One of the targets of NRF1 is the ATPase5β gene that encodes the beta subunit of complex V of the oxidative phosphorylation machinery in the mitochondria (see Figure 3A). PARIS was shown to accumulate in the brains of PD patients, with no concomitant PARIS mRNA increase, indicating regulation of PARIS at the protein level [33].

**Figure 4:**
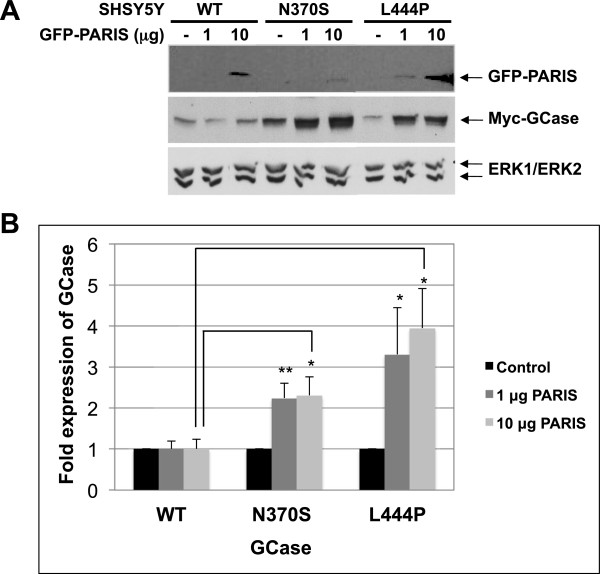
**Overexpression of PARIS leads to stabilization of mutant GCase. (A)** SHSY5Y cells stably expressing WT or N370S and L444P mutant GCase were transfected with the indicated amounts of GFP-PARIS expressing plasmid. Sixteen hours later, cell lysates were prepared and subjected to western blot analysis. The corresponding blot was interacted with anti-GFP antibody, anti-myc and anti-ERK antibodies, as a loading control. **(B)** To quantify the results, the blots were scanned and GCase intensity at each lane was divided by the intensity of ERK1/2 in the same lane. The value obtained for WT-GCase was considered as 1. The results represent the mean ± SEM of 3 independent experiments. *P < 0.05, **P < 0.01 in Student *t*-test*.*

Based on the above, and on our results showing that mutant GCase is a parkin substrate, we chose to test whether transcription of PGC1α, NRF1 and ATPase5β is repressed in GD-derived fibroblasts. The results, presented in Figure 3B, show a significant decrease in mRNA level of the three tested genes. Our results indicated the same decrease in mRNA level of PGC1α, NRF1 and ATPase5β in human dopaminergic neuroblastoma-derived cells in tissue culture (SHSY5Y), stably expressing mutant GCase [36] in comparison to their level in the same cells expressing normal GCase. These results indicated that the presence of mutant GCase leads to decreased transcription of genes regulated by PARIS, suggesting that a competition exists between PARIS and mutant GCase. To confirm these results, we tested whether increasing amounts of GFP-tagged PARIS in SHSY5Y cells, stably expressing normal or mutant GCase, leads to stabilization of mutant but not normal GCase. The results, presented in Figure 4, showed that with elevated amounts of PARIS in the cells, the level of the N370S mutant GCase increased 2-fold and the amount of the L444P mutant GCase variant increased 4-fold in comparison to the normal human GCase, expressed under the same competitive conditions.

To summarize, our results imply that mutant GCase and PARIS compete on the availability of parkin. Thus, presence of mutant GCase leads to accumulation of cytoplasmic PARIS and down-regulation of genes associated with mitochondrial biogenesis, which may lead to cell death.

### Competition between ARTS and mutant GCase

**Figure 5:**
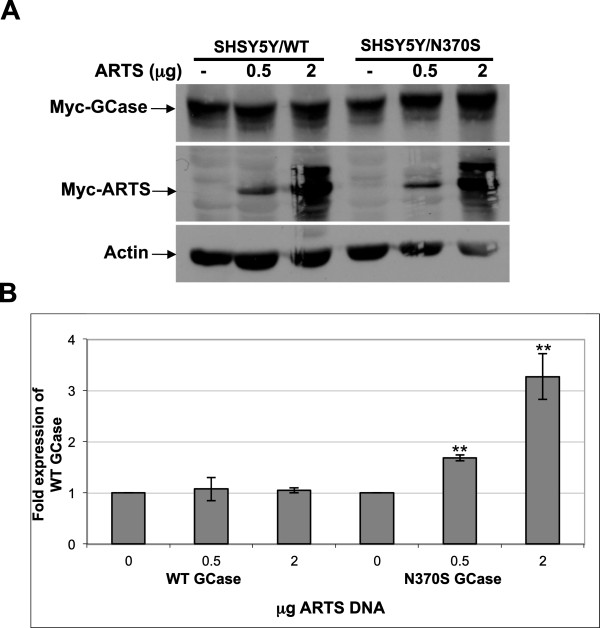
**Overexpression of ARTS lead to stabilization of mutant but not normal GCase. (A)** SHSY5Y cells stably expressing WT or N370S mutant GCase were transfected with the indicated amounts of myc-ARTS. After 48 hours cell lysates were prepared and subjected to western blot analysis. The corresponding blot was interacted with anti-myc antibody and with anti-actin antibodies as a loading control. **(B)** To quantify the results, the blots were scanned and ARTS intensity at each lane was divided by the intensity of actin in the same lane. The value obtained for non-transfected cells was considered as 1. The results represent the mean ± SEM of three independent experiments. **P < 0.005 in Student *t*-test*.*

Another parkin substrate is the pro-apoptotic protein ARTS (Apoptosis Related protein in TGFβ Signaling pathway, Sept4_i2) [34]. ARTS is a resident of the mitochondrial outer membrane, but upon apoptotic stimuli it rapidly translocates to the cytoplasm where it binds to and inhibits XIAP (E3 ubiquitin ligase X-linked inhibitor of apoptosis), leading to caspase activation and cell death [40,41]. Accumulation of ARTS in cells renders them more susceptible to apoptosis [40,42-44]. We tested a possible competition between ARTS and mutant GCase in dopaminergic cells. SHSY5Y cells, stably expressing normal or the N370S mutant GCase, were transfected with increasing amounts of ARTS-expressing plasmid. The results (Figure 5A and B) strongly indicate that overexpression of ARTS leads to accumulation of the N370S mutant but not of normal GCase.

### Mutant GCase enhances cleavage of caspase 3 and caspase 9, leading to apoptosis

**Figure 6:**
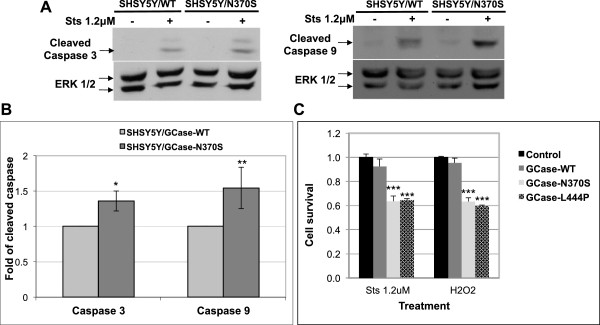
**Presence of mutant GCase increases apoptotic stimuli induced cleavage of caspase 3 and caspase 9 leading to cell death. (A)** SHSY5Y cells, stably expressing WT or N370S mutant GCase, were treated with 1.2 μM of staurosporine for 3 hours, after which cell lysates were prepared and subjected to western blot analysis. The corresponding blot was interacted with anti-cleaved caspase 3 antibody or anti-cleaved caspase 9 antibody and anti-ERK antibodies, as a loading control. **(B)** To quantify the results, the blots were scanned and intensity of cleaved caspase 3 or cleaved caspase 9 at each lane was divided by the intensity of ERK 1/2 in the same lane. The value obtained for cells expressing WT GCase was considered as 1. The results represent the mean ± SEM of five independent experiments. *P < 0.05, *P < 0.02 in Student *t*-test. **(C)** SHSY5Y cells, stably expressing either WT or N370S or L444P mutant GCase variants or naïve SHSY5Y cells (control), were treated with 1.2 μM of staurosporine for 3 hours or 1% hydrogen peroxide (H_2_O_2_) for 1 hour after which cells were subjected to XTT colorimetric assay (Biological industries, Beit Haemek, Israel). The results represent the mean ± SEM of three to five independent experiments. ***P < 0.001 in Student *t*-test*.*

Translocation of ARTS from the mitochondria to the cytosol following apoptotic stimuli leads to cleavage of caspase 3 and caspase 9 [42]. We have shown that mutant GCase competes with ARTS on the availability of parkin. Therefore, upon apoptotic stimuli and in the presence of mutant GCase, ARTS should accumulate, inducing cleavage of caspase 3 and 9. To test this assumption, cleavage of caspase 3 and 9 was assayed in staurosporine-stimulated SHSY5Y cells stably expressing normal or N370S mutant GCase. The results, presented in Figure 6A and B, indicate that the apoptotic stimuli was followed by an increase in the amount of cleaved caspase 3 and caspase 9 in SHSY5Y cells expressing N370S mutant GCase in comparison to SHSY5Y cells expressing normal GCase. We also tested whether this staurosporine-induced cleavage of caspase 3 and caspase 9 as well as another stimulus (hydrogen peroxide) lead to apoptosis. As presented in Figure 6C, SHSY5Y cells stably expressing the N370S or the L444P mutant GCase were more susceptible to apoptotic stimulus than SHSY5Y cells stably expressing the normal GCase.

Our results strongly indicate that mutant GCase leads to sensitization of cells to apoptotic stimuli and death, involving caspase cleavage.

## Discussion

In the present study we show that endogenous mutant GCase variants undergo polyubiquitination. They associate with parkin, which mediates their degradation, as shown by overexpression of normal or mutant parkin in GD-derived skin fibroblasts or by transfection of parkin shRNA into GD-derived fibroblasts. Mutant GCase variants compete with the pathogenic parkin substrates PARIS and ARTS. Based on the presented results, we hypothesize that the presence of mutant GCase in dopaminergic cells of GD patients or carriers of GD mutations leads to accumulation of pathogenic parkin substrates, which contributes to their death and to the development of PD.

Mutations in parkin, a RING domain-containing E3 ligase, are the most common cause of autosomal recessive PD [26,45]. Parkin plays a role in the ERAD of misfolded ER proteins, and it is upregulated by unfolded protein stress [46]. It induces proteasome-mediated mitophagy and degradation of mitofusins [47]. Loss of parkin activity leads to the accumulation of its pathogenic substrates PARIS and AIMP2 (aminoacyl tRNA synthetase complex-interacting multifunctional protein-2) and to α-synuclein aggregation, ultimately causing cell death [28]. PARIS is a transcription factor that undergoes parkin-mediated ubiquitination and proteasomal degradation in the cytoplasm. Its non-ubiquitinated form shuttles to the nucleus and represses the transcription of NRF1, whose target is ATPase5β. The latter encodes the beta subunit of complex V of the oxidative phosphorylation machinery in the mitochondria [33]. AIMP2 is another substrate of parkin, present in Lewy body inclusions in the substantia nigra of PD patients [48,49].

Parkin promotes clearance of depolarized mitochondria by mitophagy [50-52]. Mitophagy is the selective engulfment of mitochondria by autophagosomes and their subsequent degradation by lysosomes [53,54]. Parkin is recruited to depolarized mitochondria where it mediates the ubiquitination of the mitochondrial outer membrane proteins mitofusin 1 and 2 [51,55], thereby preventing the fusion of damaged mitochondria and targeting them for degradation by mitophagy [47,51,55,56]. Interestingly, disease-associated parkin mutants were also shown to be defective in promoting mitophagy [57].

Another parkin substrate is Miro, a component of the primary motor/adaptor complex that anchors kinesin to the mitochondrial surface. Endogenous Miro levels were significantly decreased in HEK293T cells overexpressing parkin, resulting in the release of kinesin from mitochondria and the detachment of mitochondria from microtubules [58].

Based on our results and the literature, we assume that occupation of parkin with mutant GCase affects its availability to bind other substrates, including PARIS, ARTS, mitofusins and Miro. This leads to decreased mitochondria biogenesis, prevents mitophagy and results in retention of damaged mitochondria, eventually leading to death of dopaminergic cells and to development of PD.

Phosphorylation of parkin by c-Abl and its interaction with 14-3-3eta were shown to inactivate its activity. Thus, the non-tyrosine receptor kinase c-Abl mediates dopaminergic stress or dopaminergic neurotoxin-induced tyrosine 143 phosphorylation of parkin. This modification leads to the inactivation of parkin, the subsequent accumulation of pathogenic parkin substrates, and to the eventual death of dopaminergic cells [59,60]. Interaction of parkin with 14-3-3eta was shown to negatively regulate its activity. Alpha-synuclein abrogated the 14-3-3eta-induced suppression of parkin activity [54]. However, PD-causing mutants of α-synuclein failed to activate parkin due to their inability to bind 14-3-3eta [59,60].

Since carriers of GD mutations and Type 1 GD patients are prone to develop PD, and since the common denominator between these two populations is the existence of mutant GCase, we assume that the latter is a dominant predisposing factor for development of PD. It is of note that no GCase substrate (i.e. glucosylceramide) accumulation has ever been documented in brains of carriers of GD mutations or those of Type 1 GD patients. In a recent work we have shown that transgenic expression of mutant GCase in dopaminergic/serotonergic cells of the *Drosophila melanogaster* brain leads to development of PD-like symptoms, exemplified by the death of dopaminergic cells and motor impairment (climbing disability) [61].

Parkin is not the only E3 ubiquitin ligase involved in degradation of mutant GCase variants. Other E3 ubiquitin ligases such as c-Cbl [62] and Itch [61] have been recently reported to mediate the degradation of mutant GCase variants. It is not unusual that multiple E3 ligases contribute to the stability of substrates. Thus, at least five different E3 ligases have been already documented for p53 [63-67]. It is reasonable to assume that, under different conditions and in different cells, various E3 ligases regulate the levels of mutant GCase variants by modulating its polyubiquitination and proteasomal degradation.

## Conclusions

To summarize, in the present work we show that mutant GCase variants undergo parkin-mediated degradation, a process that leads to the accumulation of pathogenic substrates such as PARIS and ARTS in cells. We assume that accumulation of such substrates in the dopaminergic cells of the brain is one of the factors that lead to their death and development of PD.

## Competing interests

The authors declare that they have no competing interests.

## Authors’ contributions

IBB and DR designed the experiments, performed them and wrote the manuscript; SL supplied a plasmid; MH designed the experiments and participated in the manuscript preparation. All authors read and approved the final manuscript.
